# Simultaneous change of wood mass and dimension caused by moisture dynamics

**DOI:** 10.1038/s41598-019-46381-8

**Published:** 2019-07-16

**Authors:** Martin Nopens, Martin Riegler, Christian Hansmann, Andreas Krause

**Affiliations:** 1 Universität Hamburg, Faculty of Mathematics, Informatics and Natural Sciences, Department Biology, Institute of Wood Science, Wood Physics, Leuschnerstr. 91 c, 21031 Hamburg, Germany; 2 Wood K plus – Competence Centre for Wood Composites and Wood Chemistry, Altenberger Straße 69, 4040 Linz, Austria

**Keywords:** Mechanical engineering, Characterization and analytical techniques

## Abstract

An investigation of simultaneous dynamic mass and length change measurement for wood is presented. In contrast to the equilibrium in moisture content and swelling and shrinking, where extensive data exists for different wood species, less information is available for the dynamics of moisture changes in direct comparison to the related dimensional changes during the sorption process. This is due to a lack of methods. A gravimetric sorption system, equipped with a high resolution camera and an automated image evaluation, is used to examine simultaneous effects of water vapour sorption dynamics and dimensional change. This method proves a strong correlation between mass and dimensional change, which is in contrast to other investigations. Equilibrium moisture content as well as swelling and shrinking data is in good agreement with literature and manual measurements. The method enables the possibility to determine swelling and shrinking values *in-situ* without disturbing the targeted climatic conditions. The system is applicable for the investigation of natural wood, modified wood, wood composites or other lignocellulosic materials.

## Introduction

All wood and other lignocellulosic materials contain a certain amount of water in their service conditions. This water exerts an important impact on many properties of the material.

Wood as hydrophilic material structure is influenced by water sorption. Depending on the surrounding conditions, wood can uptake (absorption) or release water (desorption). Within this process, swelling and shrinking take place below fibre saturation in all anatomical directions^1,2^.

In the last decades a wide range of studies have been carried out to determine wood-water-interactions and several reviews have been done^1,3,4^. Reliable data for mass and dimensional changes caused by sorption phenomena are important for the mathematical modelling of water sorption processes^5,6^. Newer studies showed that sorption data from the Forest Products Laboratory, Wood Handbook Chapter 4^7^, used for a wide range of publications and calculations, seem not to be reliable enough for scientific purposes^8,9^. Therefore, accurate sorption data are of great interest for engineering and research purposes.

The moisture content as well as swelling and shrinking values between different equilibriums are well known for different wood species^10^. Less information is available for the speed of moisture change and the related dimensional change within the sorption process. This is due to a lack of available methods, which are needed for these investigations. For this purpose, a balance with high resolution placed in a defined and controlled environment with a combined method to measure the sample dimensions is needed.

Existing methods for measuring swelling and shrinking are usually based on equilibrium state values. The dynamic determination of sample dimensions in varying climate conditions is an important technique to characterize wood, composites, modified wood or other hygroscopic materials. This information can be used to improve the basic understanding of structure and behaviour of lignocellulosic materials.

Sorption isotherms based on equilibrium moisture content (EMC) can be determined by dynamic vapour sorption (DVS) systems^11–14^. These systems often allow only one or two samples to be measured at the same time. A system to study the sorption isotherms with 100 samples in parallel at the same time was recently developed by Zelinka *et al*.^15^. More samples measured in same conditions in one experiment offer a better comparability between the results. These methods are not able to determine the sample dimensions.

Measuring systems differ depending on the level of resolution. On microscopic level, swelling was investigated by phase-contrast X-ray tomography^16^ and neutron imaging^17^. As these methods require expensive and rarely available equipment another option are macroscopic measurements. At this scale, laser displacement sensors can be used^18^. Redman *et al*.^19^ used a microbalance and scanning laser micrometres to investigate dimensional changes. Ma *et al*.^20^ investigated wood samples with an electronic balance and recorded the radial and tangential dimensional changes via CCD laser displacement sensors. One main result found by the authors was a slower moisture change compared to the dimensional change. Dimensional changes were typically recorded by laser sensors or determined by algorithms that consider the area or the width in one direction. Consequently, more advanced algorithm need to be developed, where the preliminary study of Rosner *et al*.^17^ serve as a basis. In this respect, cameras with CCD sensors for imaging combined with sensible balance systems are promising equipments^21^. With these systems, the dimensional change can be observed in several directions within one measurement leading to more reliable data.

Based on existing literature the dimensional change within the sorption process can be described in the following way. Generally, values of swelling and shrinking depend on the species^22^. When focusing on the anatomical direction of one species the dimensional change in the longitudinal direction is lower than in radial or tangential direction^1,16^. Longitudinal length change is below 1% in the hygroscopic range^23^. These dimensional changes show a linear behaviour between a moisture content of 5 and 20%^24,25^. Compared to sorption isotherms hysteresis is only shown when the dimensional change is plotted over relative humidity (r.h.)^26^. When swelling and shrinking are plotted over moisture content hysteresis disappears^27,28^.

For comparison of sorption data, the applied handling procedure is important. Only when starting with fully saturated samples a sorption isotherm can be collected, otherwise the measured data will present a scanning isotherm^29,30^. Therefore, sample preparation with water saturation is important for reliable results^29^.

The present study presents a new approach for a combined determination of mass and dimensions during a dynamic change of the environmental conditions. The method offers the investigation of water vapour sorption in combination with *in-situ* measuring dimensional change in two directions with multiple samples. The general use and possible deviations of the system are presented.

## Results

### Swelling and shrinking

**Figure 1:**
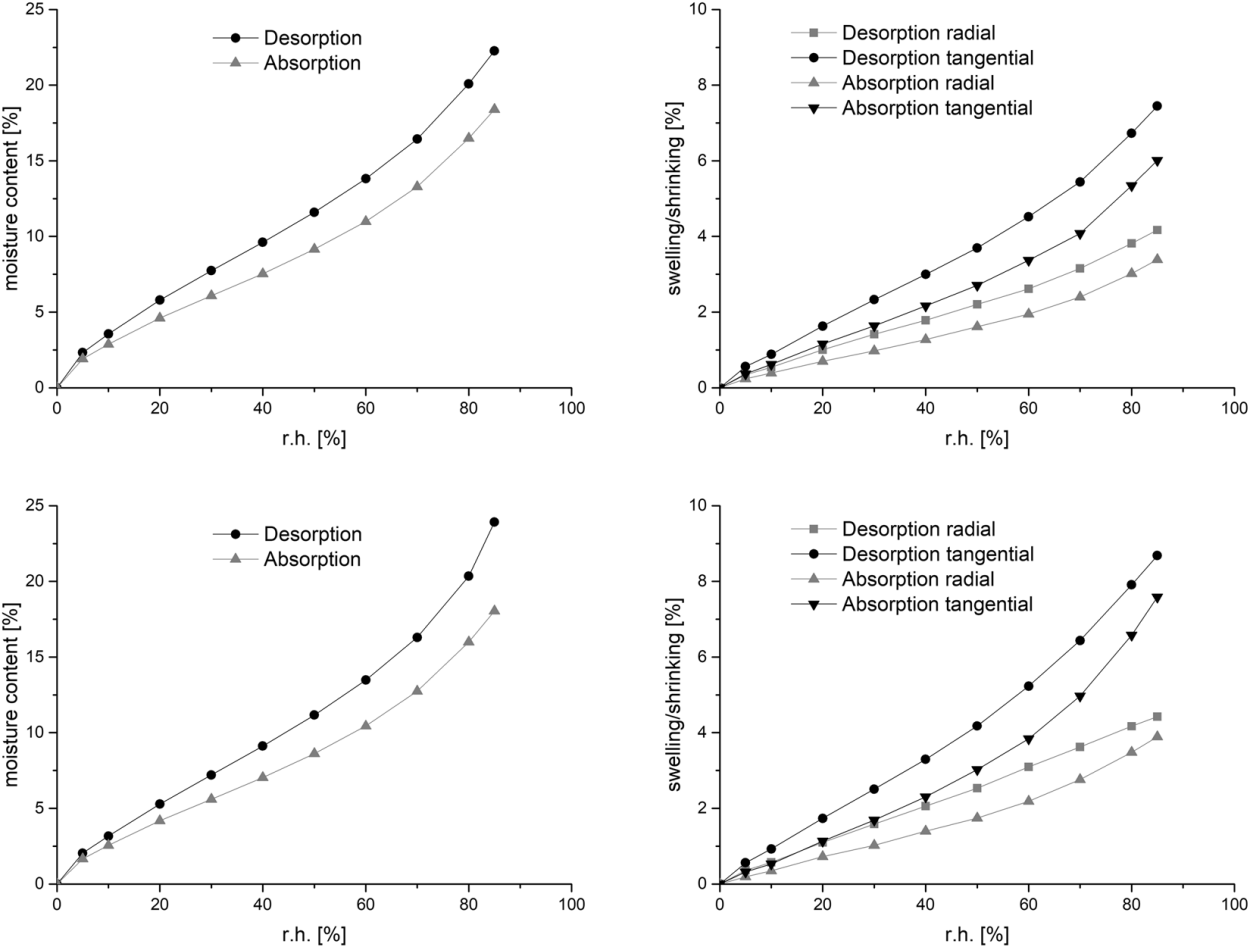
Sorption isotherms (left) and swelling and shrinking of EMC points over relative humidity (right); (top pine sample 2, down beech sample 2); measured at 20 °C; equilibrium criterion with change in mass was less than 0.01% over a 60 minutes period with 5 weighing cycles.

The method is able to record a precise mass change over relative humidity. Standard sorption isotherms for different materials in equilibrium can be measured (Fig. 1, left). With the additional setup, an *in-situ* observation of swelling and shrinking is possible in high resolution. In Fig. 1 (right) the EMC values of swelling and shrinking in dependence to relative humidity for beech and pine are shown. The change in radial direction is approximately half compared to the tangential direction. A hysteresis between absorption and desorption is clearly observable.

**Figure 2:**
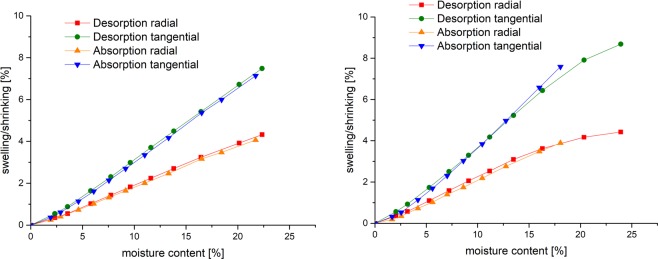
Swelling and shrinking at EMC points over moisture content (left pine sample 2, right beech sample 2).

Figure 2 displays the equilibrium state after swelling or shrinking over moisture content. The absorption and desorption curves follow the same line and hysteresis disappears. At lower moisture contents (zero to five %) absorption and desorption show a slightly non-linear behaviour. The desorption is slightly different for beech at higher moisture contents compared to pine. A slight crossing of desorption and absorption lines can be observed for beech.

**Figure 3:**
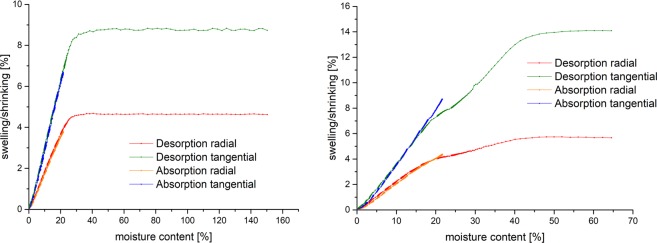
Swelling and shrinking over moisture content in the total measured sorption range; each measuring step is plotted (left pine sample 2, right beech sample 2); desorption for beech show non-linear behaviour, indicating that EMC was not reached within the sorption step.

Additionally to the evaluation of moisture and dimension at equilibrium, the dynamics of the sorption above and below fibre saturation can be analysed. The sorption dynamics of drying from fully water soaked condition to 0% and remoistening to 85% relative humidity are shown in Fig. 3. Drying does not cause a shrinking up to 40% moisture content for beech and 30% for pine, respectively. The transition between drying without shrinking and a linear relation between moisture content and shrinking is very abrupt for pine (between 32% and 28% moisture content) in contrast to beech, where transition starts at approx. 40% and ends at 20% moisture content.

Using the maximal and minimal measured dimensions of the samples, the maximum and differential swelling was calculated. Table 1 depicts the values compared to literature^31^ and manually measured samples prepared in a climate chamber. The differential swelling from the developed method is in good agreement with the compared data (Table 1) and the manual measurement.

**Table 1:** Comparison of maximum and differential swelling values between the developed method (mean values of 10 samples), literature values and manual measurements (mean values of 20 samples).

max. swelling			diff. swelling		source/method
longitudinal	radial	tangential	radial	tangential	
**pine**					
	5.6	9.3	0.19	0.31	sorption system with image evaluation
			0.15–0.19	0.25–0.36	DIN 68100
0.27	5.34	10.39	0.18	0.35	manual measurement
**beech**					
	6.0	16.4	0.15	0.41	sorption system with image evaluation
			0.19–0.22	0.38–0.44	DIN 68100
0.65	6.44	17.25	0.16	0.43	manual measurement

**Figure 4:**
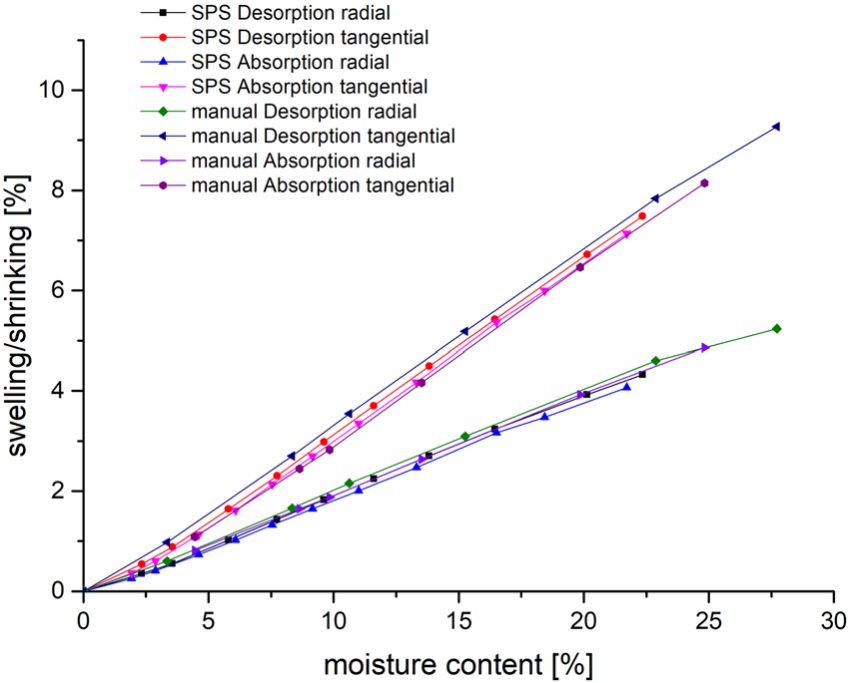
Isotherm comparison between the developed measurement system and the manual measurements of swelling and shrinking over mosisture content for pine (mean values for pine of 10 samples from SPS and 20 samples from manual measurement).

Figure 4 displays a comparison between data sets gained from the developed measurement system and manual measurements made from the identical raw material. The results show that the swelling and shrinking values of the developed measurement system are in good agreement with the manual measurements.

### Sorption dynamics

**Figure 5:**
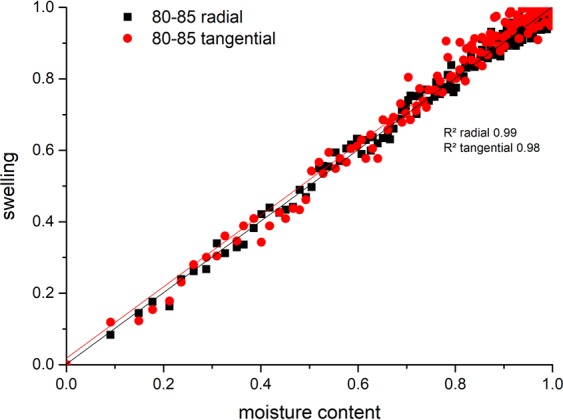
Normalized plot of swelling over moisture content in the range of 80–85% relative humidity for absorption of pine sample 2.

The simultaneous measurement of sample size and moisture content without disturbing the environment offers the opportunity to compare the dynamics of both processes. This analysis is shown in Fig. 5 for pine as example between 80% and 85% relative humidity. The values gained from the experiment for swelling (range from min 3.02% to 3.38% radial; 5.30% to 6.00% tangential) and moisture content (range from min 16.88% to max 18.44%) were normalized, plotted and a linear curve fitting was applied. There is a strong linear correlation between moisture content and swelling (R² from 0.98 to 0.99) showing that water uptake and volume change in wood occur at the same rate within the given range of relative humidity. This proves that the absorbed water causes a direct change in the sample size. In total, no significant difference between the processes of water sorption and swelling or shrinking can be seen.

**Figure 6:**
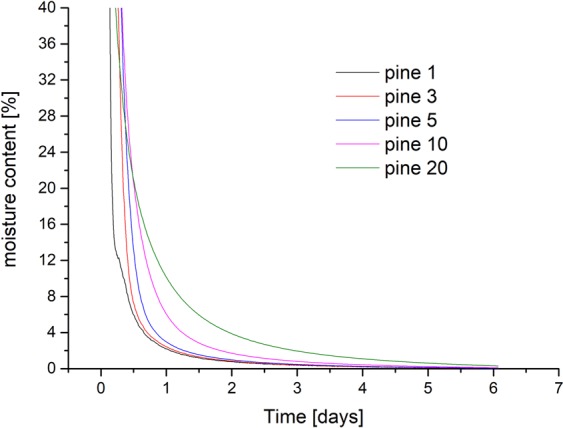
Exemplified desorption of pine with different sample thicknesses over time (number in legend corresponds to thickness in mm).

Wood samples with the square area of 20 mm × 20 mm and different thicknesses in longitudinal direction (1, 3, 5, 10, 20 mm) were measured by the developed measurement system for determining the ideal sample thickness prior to the final measurements. Desorption took place from fully saturated samples to 0% relative humidity. Afterwards, absorption was measured starting from 0% to 80% relative humidity. As an example, Fig. 6 displays the moisture content of pine during desorption over time.

Depending on the measurements with different sample thicknesses, an approximate equilibrium was calculated for the absorption and desorption speed (Table 2). Here, the original values were rounded to three decimal places. Then the first point where the difference to its successor was zero was used. After comparing this time between the different samples, the smallest sample size of 1 mm would be ideal for the measurements. A sample size of 1.5 mm thickness was chosen based on a trade-off between a fast sorption rate (i.e. for an effective measurement time) while still having a representative biological structure (i.e. cell wall geometry) of the samples.

**Table 2:** Time to reach an approximate equilibrium for beech and pine in desorption and absorption.

sample thickness (mm)	wood species	time desorption (h)	time absorption (h)
1	pine	1.26	0.24
3	pine	1.59	0.51
5	pine	1.81	0.79
10	pine	2.37	1.29
20	pine	3.29	2.08
1	beech	1.33	0.29
3	beech	1.57	0.61
5	beech	1.68	0.85
10	beech	2.44	1.50
20	beech	3.78	2.34

**Figure 7:**
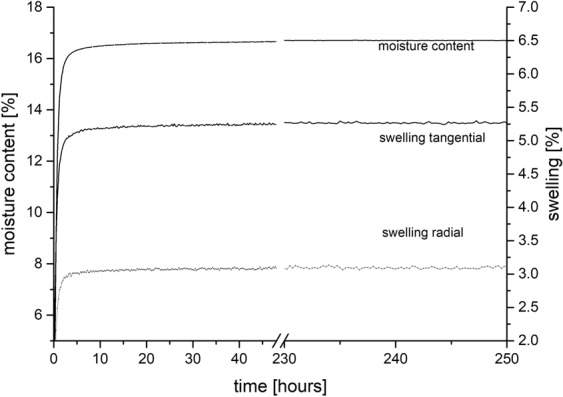
Moisture content and swelling of pine sample during absorption from 0 to 80% relative humidity over 250 h.

The measurement system showed a low long-term variability over a period of 250 h, tested for one sorption step (Fig. 7). Between 50 h and 250 h, the moisture content of pine samples showed a range (max-min) of 0.30%, the swelling in radial direction a range (max-min) of 0.65% and the swelling in tangential direction a range (max-min) of 0.43%.

## Discussion

Gravimetric data measured was reliable and highly correlates with existing literature^32–35^. Swelling and shrinking of specimens could be determined *in-situ* without disturbing the surrounding climatic conditions, which leads to more precise results. With the shown resolution, EMC points for isotherms can be determined and dynamic measurements can be realized. The results of swelling and shrinking are in agreement with literature values as well as manual measurements in a climate chamber. Dynamic data evaluation features a strong correlation between mass and length change, which is in opposite to the findings of Ma *et al*.^20^.

Holding times of water vapour sorption measurements are under critical debate^36,37^. Our equilibrium settings of mass change lower than 0.01% within 60 minutes calculated by linear regression of the last five measurement points would correspond to 0.00016% min^−1^. This value is in good agreement with the findings of Glass *et al*.^37^ who recommend the usage of 0.0003% min^−1^ as EMC criteria. This is supported by long-term measurements, where the difference between the used EMC criteria and longer sorption time is negligible (Fig. 7).

Although the presented values for absolute and differential swelling and shrinking are in good agreement with literature, several aspects need to be addressed. The beech samples probably warped with different moisture contents, which might cause an increased variation of results. On the other hand, the crossing of absorption and desorption of beech samples was also seen by Chauhan and Aggarwal^38^. A non-linear curve shape at the beginning or at the end of the isotherm was also seen for beech by Hartley & Avramidis^24^, for tropical hardwoods by Hernández^39^ and for eucalyptus by Kelsey^40^.

The non-linear curve of desorption from fibre saturation to 20% moisture content for beech could not be clarified by now (Fig. 3, right). One reason could be that some small cells within the wood are filled with water but are not cut from the outside compared to bigger cells as it was seen in magnetic resonance microimaging^41,42^. Therefore, water still needs to be transported in these regions while in other regions the cell wall is already shrinking. This would also mean that no equilibrium state has been reached within the first desorption step. Furthermore, it is possible that beech, due to more complex anatomy, causes warping or in plane movement of the sample, which disturbs the current image evaluation process. Another explanation stated by Redman *et al*.^19^ could be a cell collapse due to the fast drying process within several hours from moisture content high above fibre saturation of 20% moisture content, which was observed for eucalyptus. Additionally, pine has more homogenous pores compared to beech. Further investigations via pressure plate technique can be made to investigate this behaviour^43^. The loss of bound water at 41% moisture content inducing shrinking was also observed for birch by Almeida & Hernández^2^. In this case, the observed non-linear curve could be attributed to a species specific property.

Samples were cut from one tree to see deviations in the measuring method. In future, an investigation of several individual trees with various wood characteristics is needed to derive comprehensive knowledge for specific wood species. In the present study, technically dried timber was used for the measurements as it is mainly used in the industry. For a better fundamental knowledge, also undried wood needs to be investigated.

For the length measurements using image analysis, the definition of the threshold for CIELAB images is crucial. To avoid the influence of colour and brightness of samples, the background of the image was used to define the threshold. Additional geometrical parameters such as bending or crowning of wood samples might provide even deeper insights into anisotropic sorption processes in wood and will be included in future research, e.g. by digital image correlation.

When comparing the setup to other sorption systems, the possibility of measuring up to 11 samples at the same time is an important advantage. Additionally, with DVS systems the measurement of length changes is not feasible until today^37,44^. Alternatively, determining the volume change in a climate chamber followed by manual measuring would disturb the ambient conditions and would not allow for dynamic investigations^38,45^. The presented system overcomes these limitations.

## Conclusions

A new investigation of simultaneous dynamic mass and length change for wood is presented. High correlations between mass and dimensional change indicate a direct connection between the sorption process and swelling and shrinking. The sample size was selected based on an optimum between stable equilibrium conditions and minimum durations of single sorption steps. The method was critically evaluated for possible uncertainties. However, no significant drawbacks could be observed. Generated data is in good agreement with literature and manual measurements. It is now possible to determine swelling and shrinking values *in-situ* without disturbing the surrounding climatic conditions of the experiment. In future, image evaluation will be extended to detect annual ring deformation, bending or crown. The system can be used to investigate various wood species, modified samples, wood composites or other lignocellulosic materials.

## Material and Methods

### Measurement system and sample preparation

**Figure 8:**
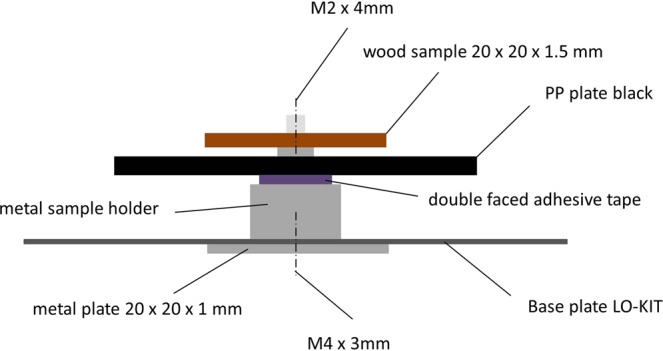
Sample holder on Large Objects Sample Tray.

Sorption isotherm and kinetics measurements where performed on a Sorption Test System (SPS) “SPSx-1µ-High-Load” by ProUmid, Germany. The resolution of the build in high resolution balance is 1 µg with a repeatability of +/−5 µg. Temperature and relative humidity are measured by a “HC2A-S Ambient Air Probe from ROTRONIC. It was equipped with a Large Objects Sample Tray (ProUmid). Eleven samples can be measured simultaneously in one experiment. An attached CCD camera (BASLER acA2040-25gc - setup by ProUmid) was used to collect individual images at every mass measurement time. A specialized sample holder for the wood specimens was developed (Fig. 8) to expose a maximum surface area of the specimens to the ambient conditions.

**Figure 9:**
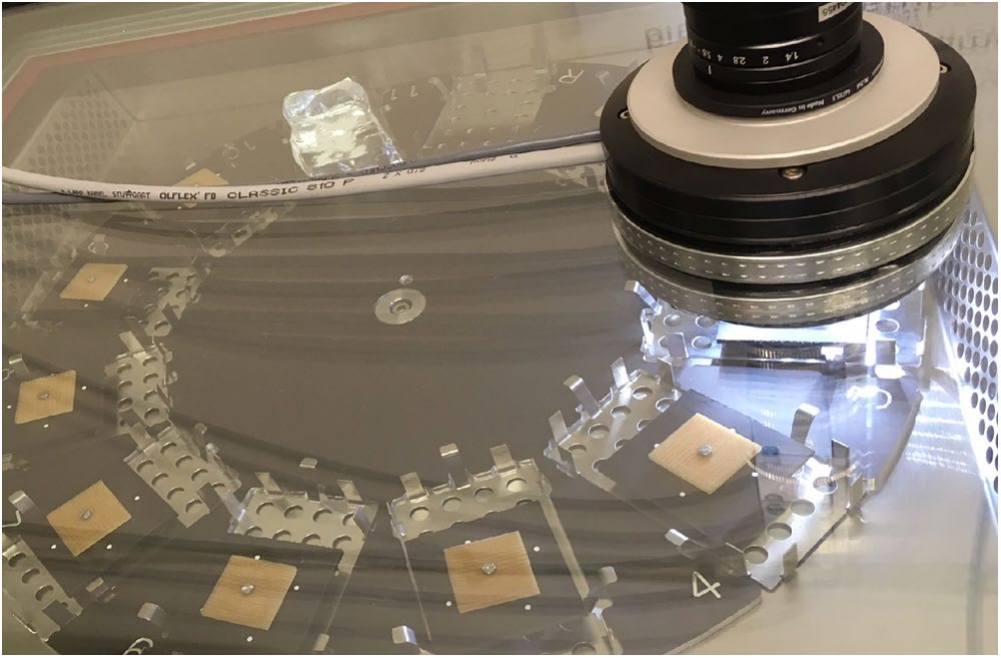
Sorption analyser with wood samples placed on sample holder within the Large Objects Sample Tray and attached CCD camera (setup by ProUmid).

Technically dried wood of European beech (*Fagus sylvatica*) and Scots pine (*Pinus sylvestris*) was used for the measurements. The total setup is shown in (Fig. 9). Samples were cut from one individual board within the adult wood (beech) and sapwood (pine) region to obtain samples with year rings parallel to one edge of the sample. In especially, wood samples with the dimension 30 × 30 × 1.5 mm (tangential, radial, longitudinal direction) were cut using a bench saw. The samples were shaped with a wood shaper to the final dimensions of 20 × 20 × 1.5 mm. Afterwards a 2 mm hole was drilled into the middle of the sample. Samples at 0% relative humidity have mean weight of 306 mg for pine and 394 mg for beech with a mean density of 0.392 g/cm³ (pine) and 0.554 g/cm³ (beech) respectively. The mean ratio between earlywood and latewood is 9.1 ± 3.6 for beech and 2.3 ± 0.6 for pine. Prior to the measurement, the samples were immersed into distillated water for one week.

The measurements were performed at 20 °C. The relative air humidity varied in steps of 5% or 10% between 85% and 0% (Table 3).

**Table 3:** Steps and set points for relative humidity.

Desorption		Adsorption	
Step	r.h. set point	Step	r.h. set point
1	85	12	5
2	80	13	10
3	70	14	20
4	60	15	30
5	50	16	40
6	40	17	50
7	30	18	60
8	20	19	70
9	10	20	80
10	5	21	85
11	0	22	90

The duration of one single conditioning step varied depending on the equilibrium time. Relative humidity was changed when the change in mass was less than 0.01% over a 60 minutes period with weighing cycles every 15 minutes. In total, minimum 5 weighing points were used to calculate the equilibrium criteria in percent as slope of a linear regression within the software automatically. Additionally a minimum cycle time of 36 h was set and the maximum time was set to 48 h. For each species, 10 samples were measured.

The moisture content *m* of the samples was calculated according to

1 $${\boldsymbol{m}}=\frac{({{\boldsymbol{m}}}_{{\boldsymbol{u}}}-{{\boldsymbol{m}}}_{0})}{{{\boldsymbol{m}}}_{0}}\ast 100\,[ \% ]$$

were, *m*_*u*_ represents the current mass of the sample and *m*_0_ represents the mass of the dry sample at 0% relative humidity. Equilibrium moisture content are mean values for the whole sample determined in an “equilibrium condition”, which was defined as a mass change lower than 0.01% within 60 min.

For the measurement with different sample thicknesses, wood pieces with 20 × 20 mm in square and different longitudinal length (1, 3, 5, 10 and 20 mm) were cut with a bench saw. Afterwards the samples were immersed in water prior to the measurement in the sorption chamber.

Manual measurements of beech and pine samples (20 × 20 × 10 mm; 20 pieces each) were performed with a caliper. The samples were conditioned in Binder KBF 720 climate chamber. The duration for each step was set to a minimum of 10 days prior to the measurement at different relative humidity steps according to an in-house handling procedure (detail information can be found in the supplementary material). Radial, tangential and longitudinal values were collected for both species based on the same material as the digital evaluated samples. The measurements of mass and length change were performed outside of the climate chamber directly after removal from climate zone. For the dry state - drying was performed within a drying oven Heraeus T6120 at 103° for 2 days. Sample mass was recorded with a Balance Sartorius LP3200D (Resolution 0.001 g, Reproducibility +/−0.001 g) and dimensions were recorded with a Caliper Mitutoyo 293-521-30 Micrometer (resolution of 0,001 mm).

### Image analysis

#### Image pre-processing

Depending on the total duration of the measurement, up to 3,329 images were taken for each sample. Images (RGB) had a size of 2,046 × 2,046 pixels and a resolution of 0.018 mm/pixel. According to the Nyquist–Shannon sampling theorem, the maximum detectable accuracy of objects within the image is 0.036 mm. As relative dimensional changes are of main interest in the present study, the focus was laid on a high reproducibility of the imaging with regard to exposure, camera settings, focus area as well as sample preparation and mounting.

For the analysis of images, scripts were developed using MATLAB R2017b and the inbuilt image processing toolbox. Images were chronologically sorted and imported into MATLAB. Then RGB images were converted into CIELAB colour space. To detect the edges of the samples, images were binarized using the threshold L = 1.821/17.536, a = −2.656/7.803, b = −11.501/3.916, to subtract the background within the image. “Artefacts” (connected objects with a maximum size of 7,000 pixels) outside of the edges of the sample were removed and “holes” (background pixels within the edges of the sample) within the sample were filled. Afterwards images were rotated to align the sample parallel to the edge of the image using a rotation matrix. Additionally, the tangential direction of all samples was aligned horizontally.

#### Calculation of geometrical parameters

**Figure 10:**
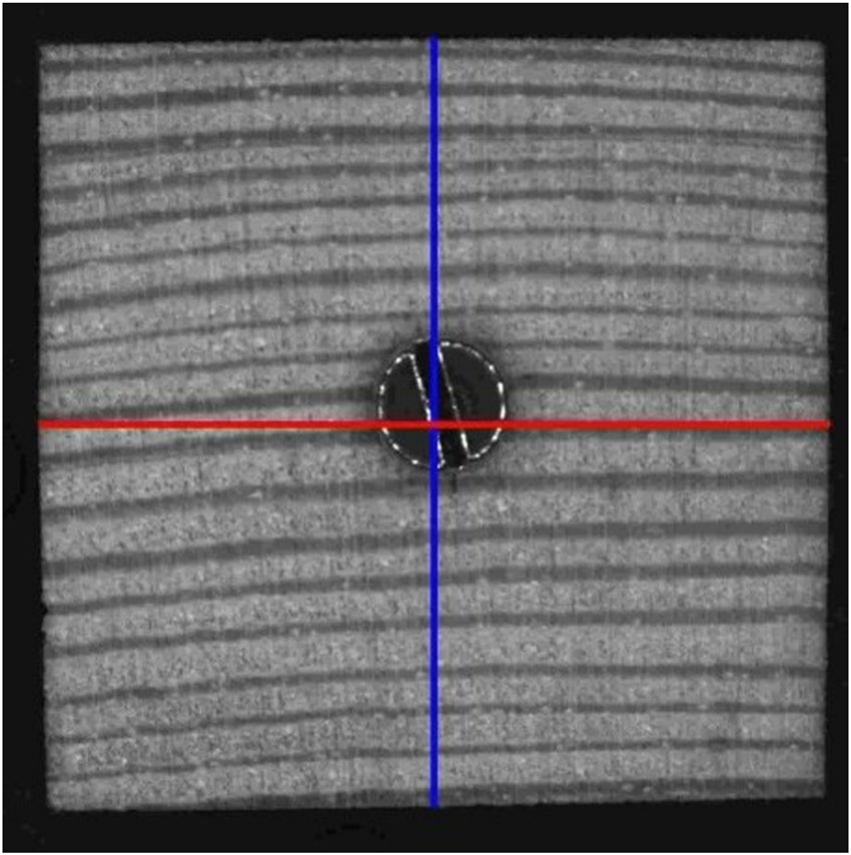
Exemplified image of a pine sample with length measurements indicated by lines in radial (blue) and tangential (red) direction.

Parameters were calculated using matrices of the binary or grayscale images. Figure 10 shows an exemplified grayscale image of a pine sample with lines indicating the positions of measurements. The width of the sample in radial direction *w*^(*cr*)^ was measured by summing up nonzero entries of the binary matrix *I* that indicate the sample across the centre of the sample using the arithmetic mean of a set of *f* columns, consisting of 81-neighbored columns (1.473 mm) by

2 $${{\boldsymbol{w}}}^{({\boldsymbol{cr}})}=\frac{{\sum }_{{\boldsymbol{j}}={\boldsymbol{c}}-{\boldsymbol{h}}}^{{\boldsymbol{c}}+{\boldsymbol{h}}}\,{\sum }_{{\boldsymbol{i}}=1}^{{\boldsymbol{m}}}\,{{\boldsymbol{I}}}_{{\boldsymbol{i}},{\boldsymbol{j}}}}{{\boldsymbol{f}}},$$

where *c* indicates the central column of the sample, *m* the number of rows in *I* and *h* about half of the set of columns *f* by

3 $${\boldsymbol{h}}=\frac{{\boldsymbol{f}}-1}{2}.$$

Analogous, *w*^(*ct*)^ was calculated in tangential direction by

4 $${{\boldsymbol{w}}}^{({\boldsymbol{ct}})}=\frac{{\sum }_{{\boldsymbol{i}}={\boldsymbol{r}}-{\boldsymbol{h}}}^{{\boldsymbol{r}}+{\boldsymbol{h}}}\,{\sum }_{{\boldsymbol{j}}=1}^{{\boldsymbol{n}}}\,{{\boldsymbol{I}}}_{{\boldsymbol{i}},{\boldsymbol{j}}}}{{\boldsymbol{f}}},$$

where *r* indicates the central row of the sample and *n* the number of columns in *I*.

The differential swelling and shrinking values where calculated by dividing the maximum length change in percent in the middle of the sample in each direction by the maximum mass when the sample began to shrink (in percent). In especially, this point in time was taken from the plot of length change versus moisture content (Fig. 3), whereas samples of beech began to shrink at 40% moisture content and samples of pine began to shrink at 30% moisture content.

For comparing swelling and shrinking against the moisture content for each sorption step, the values were normalized. In especially, the maximum differential swelling value of one sorption step was set to one and the lowest differential swelling value was set to zero.

## Supplementary information

Supplementary data - Simultaneous change of wood mass and dimension caused by moisture dynamics – Nopens Riegler Hansmann Krause

Raw data - Simultaneous change of wood mass and dimension caused by moisture dynamics – Nopens Riegler Hansmann Krause

## Data Availability

Data generated during this study are included in Supplementary Information files.
