# Supplementary material for: Simultaneous change of wood mass and dimension caused by moisture dynamics
Source: Sci Rep. 2019 Jul 16;9:10309. doi: 10.1038/s41598-019-46381-8 (PMC6635364; doi:10.1038/s41598-019-46381-8)

**Supplementary data related to the article**

**Simultaneous change of wood mass and dimension caused by moisture dynamics**

by Martin Nopens*^1^, Martin Riegler^2^, Christian Hansmann^2^, Andreas Krause^1^

^1^ Universität Hamburg, Faculty of Mathematics, Informatics and Natural Sciences, Department Biology, Institute of Wood Science, Wood Physics, Leuschnerstr. 91 c, 21031 Hamburg, Germany

^2^ Wood K plus – Competence Centre for Wood Composites and Wood Chemistry, Altenberger Straße 69, 4040 Linz, Austria

Plot of total measurement for pine (top) and beech (bottom) of sample 2 with mass change based on the lowest net weight and relative humidity (read) on y-axis


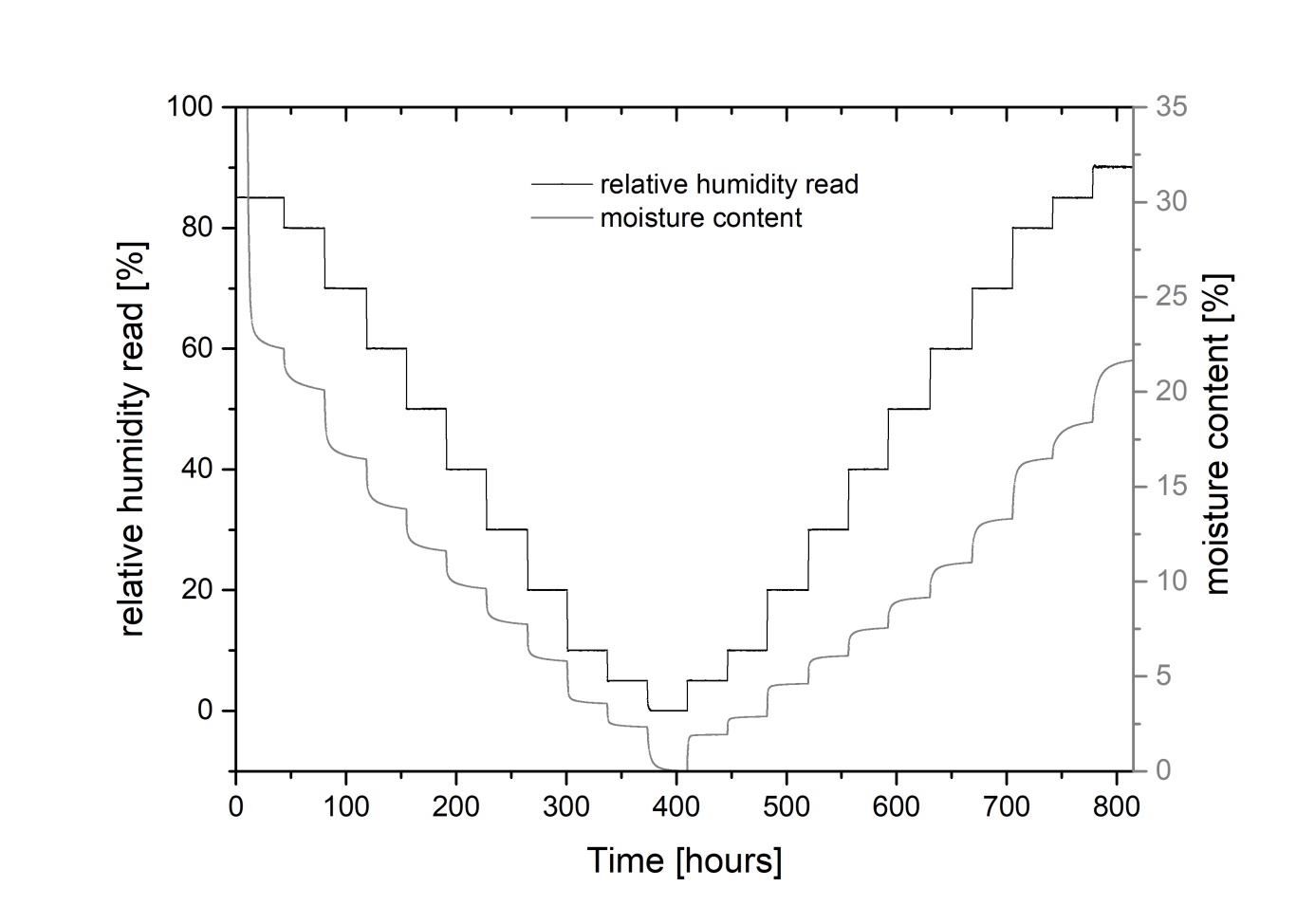


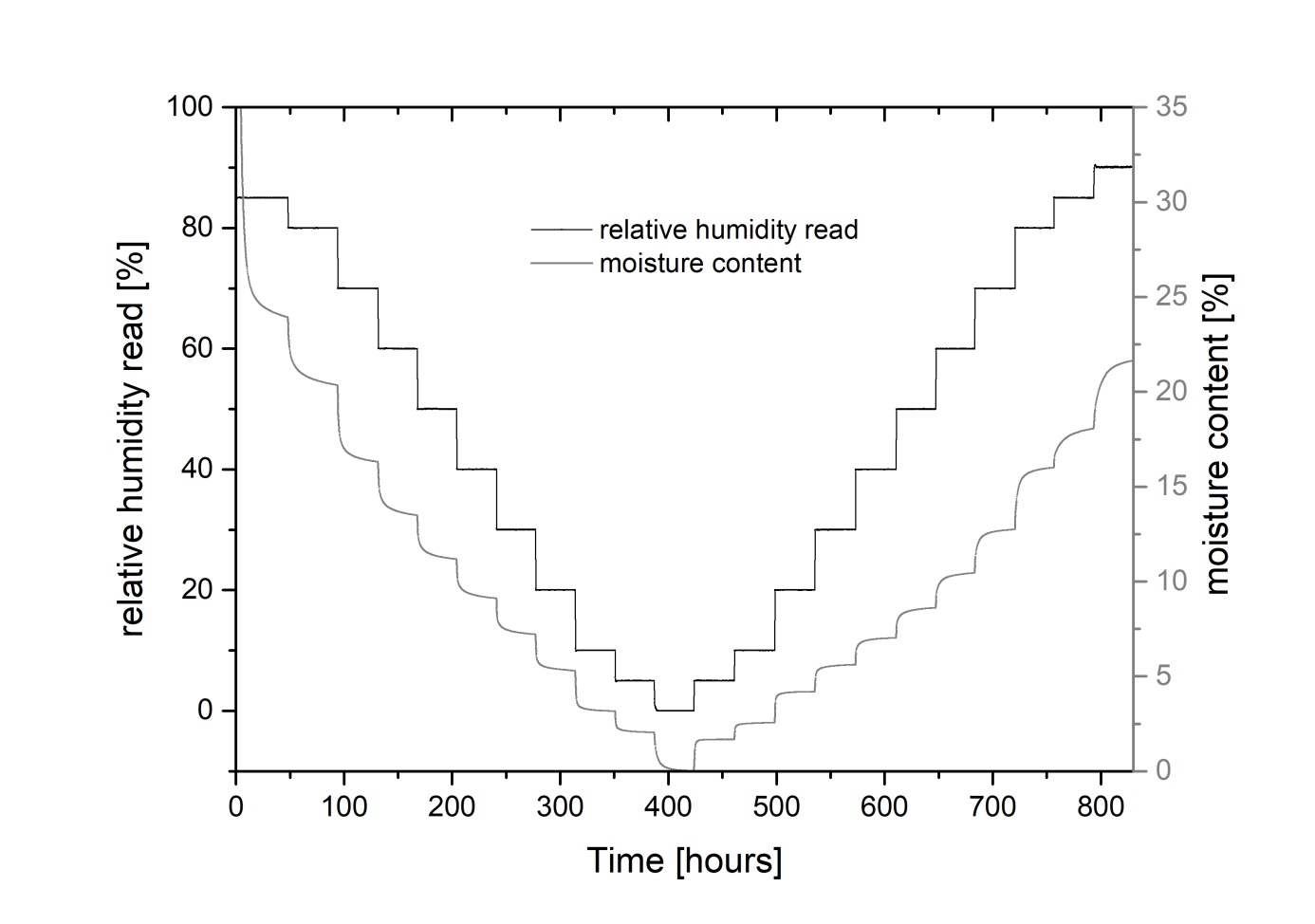


Relative humidity setpoints of manual comparison measurements

| Step | T in °C | r.h. setpoint |
| --- | --- | --- |
| 1 | 30 | 10 |
| 2 | 20 | 35 |
| 3 | 20 | 45 |
| 4 | 20 | 65 |
| 5 | 20 | 85 |
| 6 | 20 | 95 |
| 7 | 20 | water |
| 8 | 20 | 95 |
| 9 | 20 | 85 |
| 10 | 20 | 65 |
| 11 | 20 | 45 |
| 12 | 20 | 35 |
| 13 | 30 | 10 |
| 14 | 103 | 0 |
| 15 | 25 | 35 |

Main sample details

| beech at relative humidity 0% | | | | | | |
| --- | --- | --- | --- | --- | --- | --- |
| Number | sample mass in mg | tangential length (mm) | radial length (mm) | longitudinal length (mm) | Volume in mm³ | density in g/cm³ |
| 1 | 403,787434 | 21,9252525 | 21,949719 | 1,51 | 726,69 | 0,556 |
| 2 | 405,276448 | 21,8017957 | 21,855219 | 1,52 | 724,25 | 0,560 |
| 3 | 386,817604 | 21,9021324 | 21,874972 | 1,46 | 699,5 | 0,553 |
| 4 | 395,611376 | 21,6810325 | 21,950393 | 1,51 | 718,62 | 0,551 |
| 5 | 393,698215 | 21,8949495 | 21,836813 | 1,49 | 712,39 | 0,553 |
| 6 | 385,122448 | 21,7829405 | 21,769697 | 1,49 | 706,57 | 0,545 |
| 7 | 398,41947 | 21,7907969 | 21,856117 | 1,49 | 709,63 | 0,561 |
| 8 | 399,032354 | 21,827385 | 21,794388 | 1,52 | 723,09 | 0,552 |
| 9 | 381,129175 | 21,7537598 | 21,929068 | 1,45 | 691,71 | 0,551 |
| 10 | 395,014018 | 21,5838384 | 21,830527 | 1,51 | 711,49 | 0,555 |
| mean | 394,390854 | 21,7943883 | 21,864691 | 1,495 | 712,39 | 0,554 |
| pine at relative humidity 0% | | | | | | |
| 1 | 301,576197 | 21,8603816 | 21,823569 | 1,62 | 772,86 | 0,390 |
| 2 | 303,663641 | 21,6936027 | 21,771717 | 1,64 | 774,58 | 0,392 |
| 3 | 301,902413 | 21,7777778 | 21,766779 | 1,63 | 772,67 | 0,391 |
| 4 | 306,940764 | 21,6482604 | 21,775084 | 1,62 | 763,66 | 0,402 |
| 5 | 310,49192 | 21,7705948 | 21,774186 | 1,68 | 796,38 | 0,390 |
| 6 | 318,468332 | 21,6011223 | 21,68642 | 1,7 | 796,37 | 0,400 |
| 7 | 300,86714 | 21,7506173 | 21,767003 | 1,63 | 771,72 | 0,390 |
| 8 | 303,71657 | 21,7916947 | 21,797082 | 1,65 | 783,74 | 0,388 |
| 9 | 305,563986 | 21,7791246 | 21,83569 | 1,65 | 784,68 | 0,389 |
| 10 | 307,773411 | 21,7239057 | 21,691134 | 1,66 | 782,22 | 0,393 |
| mean | 306,096438 | 21,7397082 | 21,768866 | 1,648 | 779,89 | 0,392 |

|  | early wood [mm] | | late wood [mm] | | early/late wood ratio | | sample quantity |
| --- | --- | --- | --- | --- | --- | --- | --- |
|  | mean | standard deviation | mean | standard deviation | mean | standard deviation |  |
| beech | 3,6333 | 0,6132 | 0,4596 | 0,1744 | 9,1361 | 3,638 | 5 |
| pine | 0,7758 | 0,144 | 0,3588 | 0,0867 | 2,2654 | 0,5794 | 5 |

Pictures of beech and pine at 0% moisture content (sample 2 of each measurement); Picture number 1695 for beech and 1640 for pine


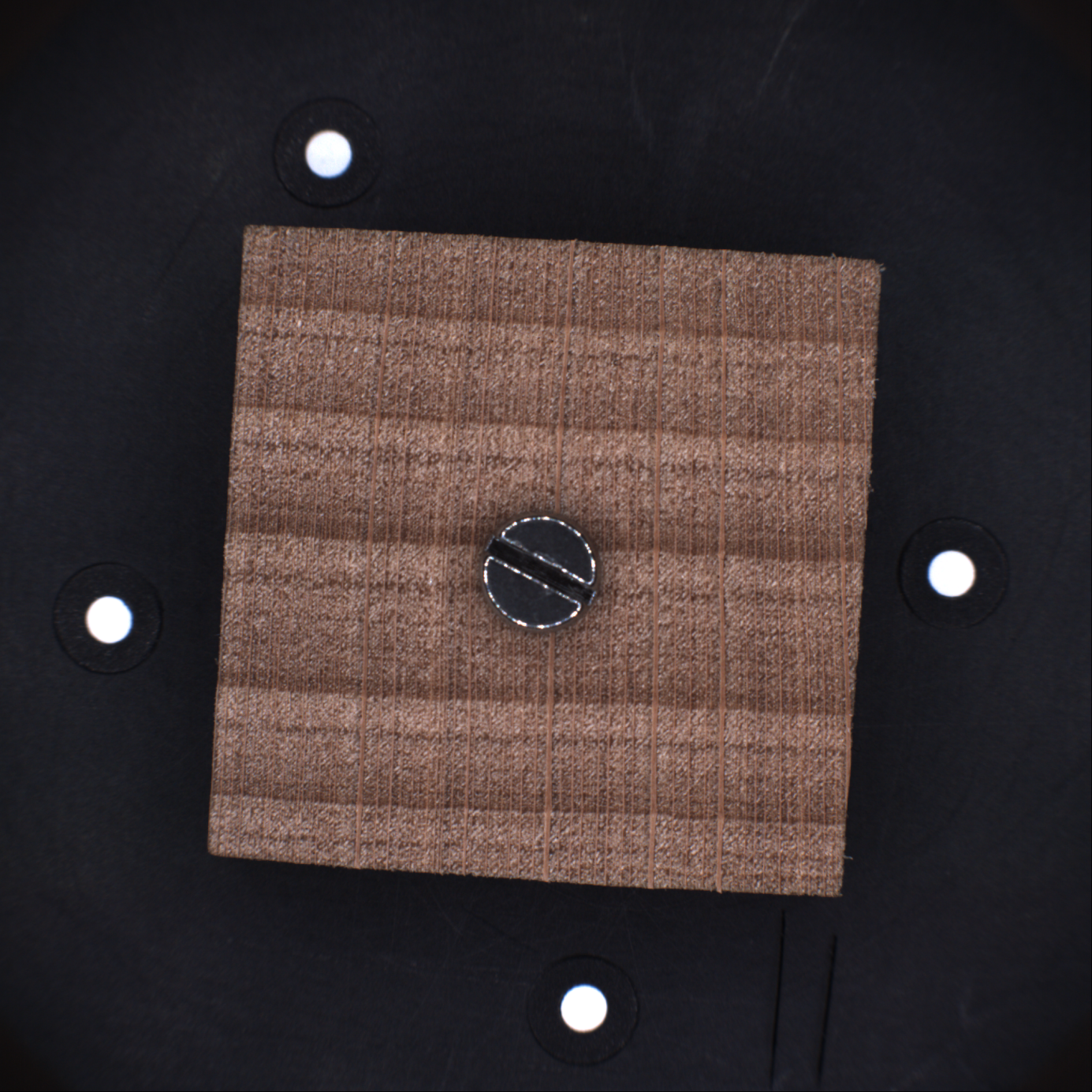


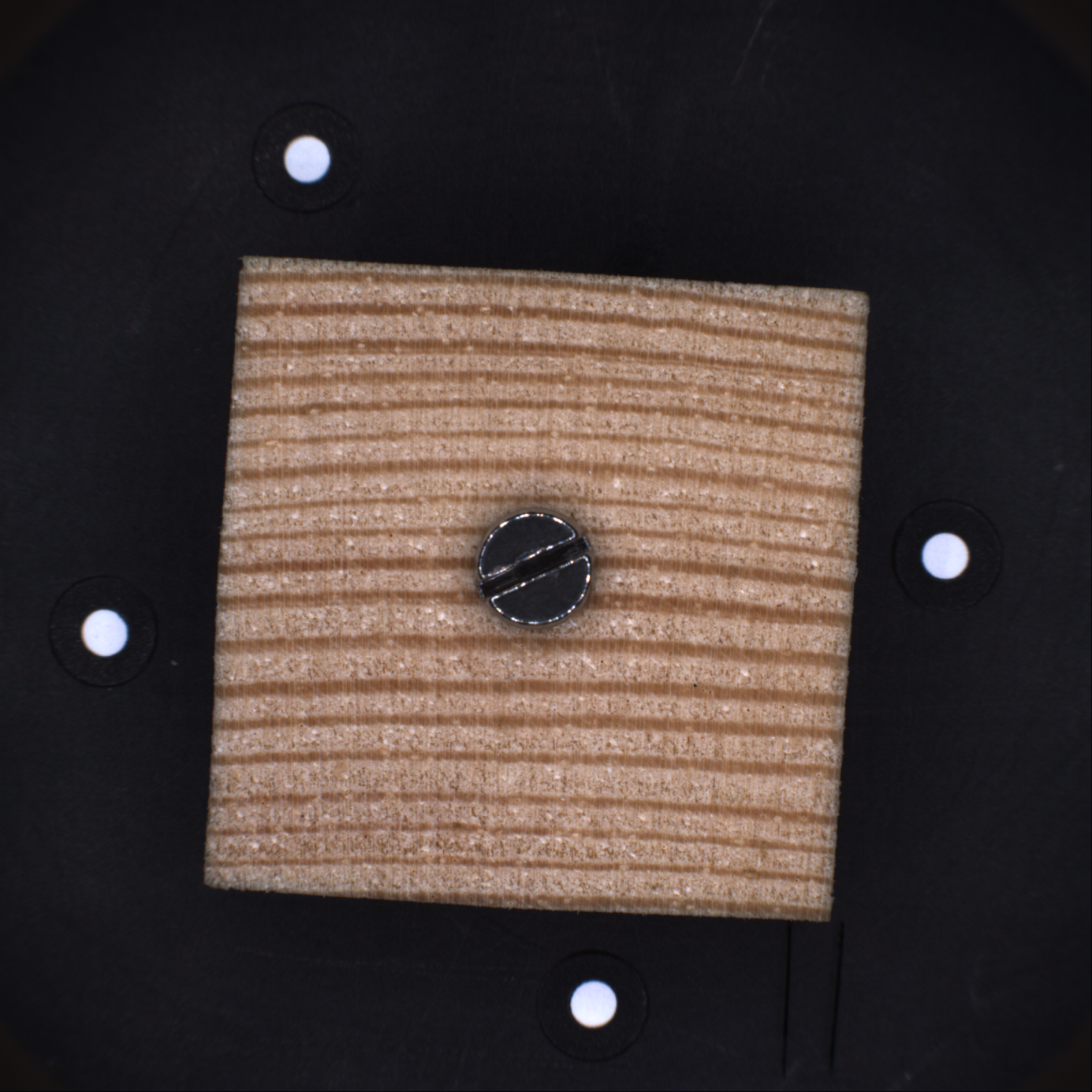

Supplement: Supplementary file 1 — Supplementary data - Simultaneous change of wood mass and dimension caused by moisture dynamics – Nopens Riegler Hansmann Krause [file 41598_2019_46381_MOESM1_ESM.docx]
